# Electronically tunable extraordinary optical transmission in graphene plasmonic ribbons coupled to subwavelength metallic slit arrays

**DOI:** 10.1038/ncomms12323

**Published:** 2016-08-08

**Authors:** Seyoon Kim, Min Seok Jang, Victor W. Brar, Yulia Tolstova, Kelly W. Mauser, Harry A. Atwater

**Affiliations:** 1 Thomas J. Watson Laboratory of Applied Physics, California Institute of Technology, Pasadena, California 91125, USA; 2 School of Electrical Engineering, Korea Advanced Institute of Science and Technology, Daejeon 34141, Korea; 3 Kavli Nanoscience Institute, California Institute of Technology, Pasadena, California 91125, USA

## Abstract

Subwavelength metallic slit arrays have been shown to exhibit extraordinary optical transmission, whereby tunnelling surface plasmonic waves constructively interfere to create large forward light propagation. The intricate balancing needed for this interference to occur allows for resonant transmission to be highly sensitive to changes in the environment. Here we demonstrate that extraordinary optical transmission resonance can be coupled to electrostatically tunable graphene plasmonic ribbons to create electrostatic modulation of mid-infrared light. Absorption in graphene plasmonic ribbons situated inside metallic slits can efficiently block the coupling channel for resonant transmission, leading to a suppression of transmission. Full-wave simulations predict a transmission modulation of 95.7% via this mechanism. Experimental measurements reveal a modulation efficiency of 28.6% in transmission at 1,397 cm^−1^, corresponding to a 2.67-fold improvement over transmission without a metallic slit array. This work paves the way for enhancing light modulation in graphene plasmonics by employing noble metal plasmonic structures.

## Abstract (web summary)

Graphene-plasmon optical modulators with broadly tunable operating frequencies are sought for photonic applications. Here, Kim *et al.* demonstrate tunable mid-infrared transmission that utilizes resonant absorption in graphene ribbons to modulate the extraordinary optical transmission effect in metallic slit arrays.

## Graphical abstract

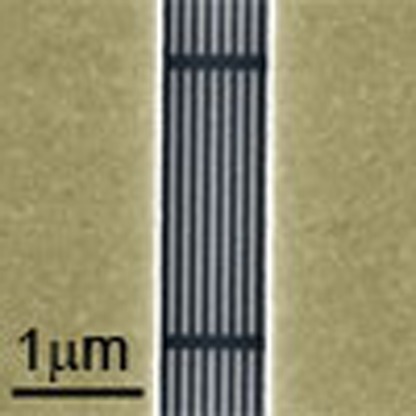

Significant efforts have been made in the past 5 years to create graphene plasmon-based optical modulators that function from THz to mid-infrared frequencies. These devices have exploited the unique plasmon dispersion relation of graphene, which exhibits optical modes with high-confinement factors^1^,^2^,^3^,^4^,^5^,^6^, and that are electrostatically tunable^7^,^8^. Typically, these devices have been based on geometries that employ resonant absorption in graphene nanostructures that have been patterned to confine plasmonic modes that can be electrostatically tuned to control the intensity and frequency of either optical absorption or emission^9^,^10^,^11^,^12^,^13^,^14^,^15^,^16^,^17^,^18^,^19^,^20^,^21^,^22^. However, the single-layer atomic thickness and low free carrier density of graphene has limited the efficiencies of such modulators, especially at mid-infrared frequencies, where the oscillator strength of the graphene plasmonic modes is low. A number of strategies have been adopted to overcome these difficulties, including using ionic gel or chemical doping to increase the carrier density of the graphene sheet^12^,^13^,^23^,^24^, or carefully controlling the substrate to include a metallic back reflector, which creates additional optical resonances that enhance field intensities at the graphene plasmonic ribbons (GPRs)^18^,^19^,^20^,^23^, and thus enhance absorption. While those techniques can have theoretical modulation efficiencies of 100%, the use of ionic gels places significant restraints on the applicability, switching speeds, and durability of such devices, and the use of metallic reflectors forces those devices to be used in reflection geometries. In contrast to the reflective-type graphene plasmonic modulators, a strategy of using electrostatically tunable graphene plasmons to modulate transmitted light with near-unity efficiency has not yet been reported, to the best of our knowledge. The creation of such a device would have widespread applications in optoelectronic devices such as mid-infrared spatial light modulators, or linear signal processing^25^,^26^,^27^.

In this paper, we report an approach to use graphene plasmonic modes for light modulation in a transmission geometry that satisfies the above conditions—high modulation efficiency, at carrier densities accessible with electrostatic gating. Our modulator design is based on a triple resonant structure, where the plasmonic resonances in GPR are matched to a dielectric substrate Fabry–Perot resonance, and also to the optical resonances in a subwavelength metallic slit array, which is designed to exhibit extraordinary optical transmission (EOT) in the mid-infrared. In full-wave simulations, the proposed structure shows 95.7% modulation efficiency in transmission. We measured a mid-infrared transmission modulation efficiency of 28.6% at 1,397 cm^−1^, which is 2.67 times higher than that measured for an equivalent GPRs-only structure on the same supporting dielectric structure.

## Results

### Device geometry and light modulation mechanism

**Figure 1:**
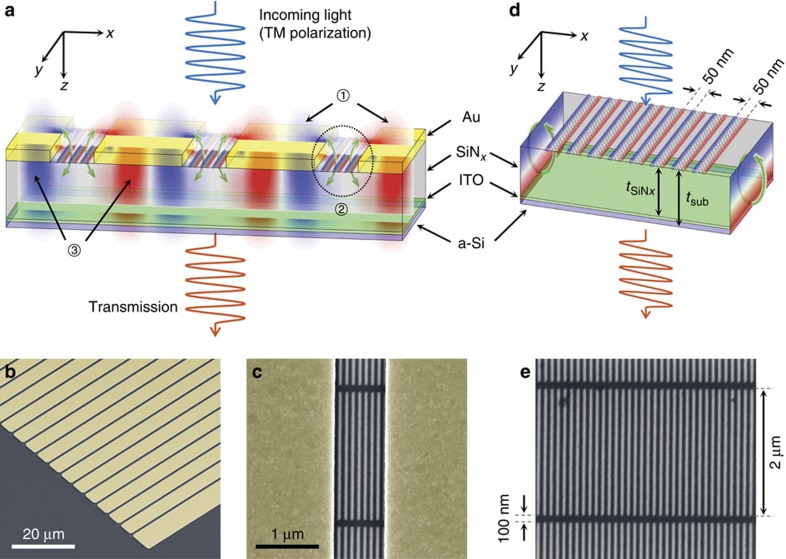
Device and working mechanism. (**a**) Schematic of the graphene plasmonic ribbons (GPRs) coupled to subwavelength metallic slit array. Under normal operation (that is, no GPRs), transverse magnetic (TM) polarized incoming light induces surface plasmons on the top metal surface (1) that tunnel through the subwavelength metallic slits (2), exciting surface plasmons (3) on the bottom metal surface. The surface plasmons on the bottom metal surface are diffracted by the periodic structure and radiate into free space with an enhanced intensity. The role of the GPRs inside the subwavelength metal slits is to block the coupling channel (4), and leading to a suppression of the extraordinary optical transmission effect. In this figure, the overlapping field distribution depicts Re(*E*_*z*_) for the surface plasmons, and the scale is adjusted to fit the schematic. SEM images (false colour) of (**b**) the subwavelength metallic slit array and (**c**) the GPRs inside the subwavelength metallic slit fabricated on SiN_*x*_ membrane. (**d**) Schematic of a transmission-type bare GPR modulator. Field distributions on the side walls correspond to Re(*E*_*x*_) showing the Fabry–Perot resonance in the substrate. (**e**) SEM image of bare GPRs fabricated on SiN_*x*_ membrane. The fabricated GPRs form a mesh to prevent electrical disconnection with 2 μm length and 100 nm bridge. The width and the gap of the GPRs are both 50 nm.

Figure 1a shows the mechanism of the proposed device. In extraordinary optical transmission, incoming light is scattered by the periodic structure into surface plasmons on the top metal surface. The surface plasmons then tunnel through the subwavelength metallic slits and excite surface plasmons on the bottom metal surface. The surface plasmon on the bottom metal surface subsequently re-radiates into free space, resulting in a transmitted diffraction peak with a strong intensity at the extraordinary optical transmission resonance frequency^28^,^29^,^30^,^31^,^32^,^33^. The subwavelength metallic slits play a pivotal role in EOT via optical coupling between the surface plasmons on the top and bottom metal surfaces. In our modulator, GPRs are placed in the subwavelength metallic slits to modulate the EOT resonant coupling. This is accomplished by electrostatically tuning the plasmonic resonances in GPRs to match the resonant frequency of the EOT. When matched, the electric fields in the subwavelength metallic slits give rise to large plasmonic resonance in the GPRs, which leads to blocking the coupling channel for the EOT resonance. As a result, a strong suppression of EOT occurs. To demonstrate this modulation mechanism, we fabricated subwavelength metallic slit array structures with GPRs on SiN_*x*_ membranes, as shown in Fig. 1b,c. In Fig. 1c, the dark stripes denote the GPRs, and the bright bar corresponds to the etched region that defines the GPRs. Figure 1d shows the schematic of the equivalent GPRs-only structure on the same supporting dielectric structure as a control sample, and the SEM image of the fabricated device is shown in Fig. 1e.

### Substrate geometry optimization

**Figure 2:**
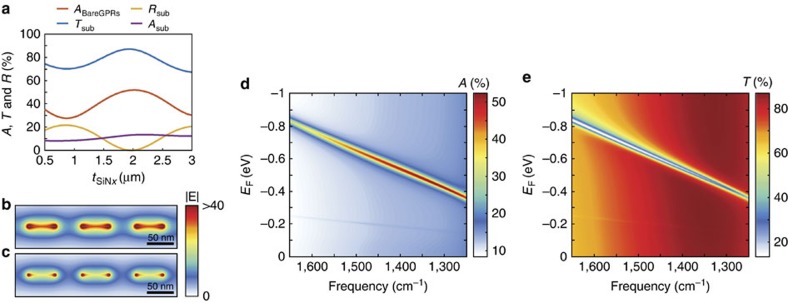
Graphene plasmonic ribbons. (**a**) The red line indicates absorption in the bare graphene plasmonic ribbons (GPRs) device depending on the SiN_*x*_ thickness (*t*_SiN*x*_) with the graphene Fermi level position *E*_F_=−0.465 eV. The blue, yellow and purple lines correspond to transmittance, reflectance and absorption, respectively, depending on the SiN_*x*_ thickness through the SiN_*x*_/ITO/a-Si substrate without the GPRs. Total electric field distributions around the bare GPRs at the *E*_F_=−0.465 eV with the SiN_*x*_ thickness of (**b**) 2.02 μm and (**c**) 0.87 μm. (**d**) Absorption and (**e**) transmittance in the bare GPRs device as a function of frequency and *E*_F_ with *t*_SiN*x*_=2 μm.

To achieve electronically tunable transmission, the substrate must be transparent at the operating frequency, yet also allow for electrostatic gating. In addition, the substrate will support Fabry–Perot-type resonances, which can lead to constructive or destructive interference effects that modify the electric field intensities on the top surface and thus the absorption in the GPRs. To satisfy these constraints, we use a SiN_*x*_ membrane with a DC conducting indium tin oxide (ITO, 4nm)/a-Si (60 nm) contact on the bottom side of the membrane, as shown in Fig. 1a. To optimize the substrate thickness for maximizing absorption in GPRs, we performed full-wave simulations for an array of bare 50 nm wide GPRs, varying the SiN_*x*_ thickness. The calculations were performed at *λ*^−1^=1,340 cm^−1^, which is the resonant frequency of 50 nm GPRs for *E*_F_=−0.465 eV, and a graphene carrier mobility (*μ*_h_) of 15,000 cm^2^ V^−1^ s^−1^ was assumed for the graphene sheet^8^. Here the negative sign of *E*_F_ denotes that the graphene is hole doped. As shown in Fig. 2a, the transmission spectrum exhibits a Fabry–Perot resonance that depends on the SiN_*x*_ thickness (*t*_SiN*x*_), and leads to variation in plasmonic absorption by GPRs. In contrast to GPR reflection modulators that achieve maximum absorption at *t*_sub_=*λ*/4*n*_sub_ (refs 19, 20), the absorption for transmission modulators has a maximum at *t*_sub_=*λ*/2*n*_sub_ with *t*_SiN*x*_=2.02 μm, and the minimum occurs at *t*_sub_=*λ*/4*n*_sub_ with *t*_SiN*x*_=0.87 μm (*t*_sub_: substrate thickness, *t*_SiN*x*_: SiN_*x*_ thickness, *n*_sub_: effective refractive index of substrate). Considering this structure as a Fabry–Perot cavity, the maximal absorption point corresponds to a transmission resonance in the forward direction (also see Supplementary Fig. 1). This effect arises from the zero phase shift for light reflected from the bottom a-Si/air interface. These reflected waves constructively interfere with incident light when the reflection path length is 2*t*_sub_=*λ*/*n*_sub_, which corresponds to 2*π* in terms of the phase difference. As a result, a standing wave is formed in the substrate with a maximum on the surface, which leads to enhanced absorption in GPRs. We also observe that the near-field intensities around GPRs are enhanced at resonance, as shown in Fig. 2b,c. To further characterize the device, we calculate the spectral absorption as a function of graphene Fermi level position (*E*_F_) for a 2 μm layer of SiN_*x*_, as shown in Fig. 2d. In the absence of substrate resonances, a higher-doping level, by itself, leads to strong oscillator strength in the GPRs and thus enhances plasmonic absorption^13^,^14^,^15^. However, in this device the absorption is strongest at *λ*^−1^=1,340 cm^−1^, which occurs not for maximal doping level but when the plasmonic resonance in GPRs and substrate resonance are matched, as shown Fig. 2d. As a result, transmission declines along the plasmonic absorption in GPRs, as shown in Fig. 2e.

### Theoretical modulation of coupled structure

**Figure 3:**
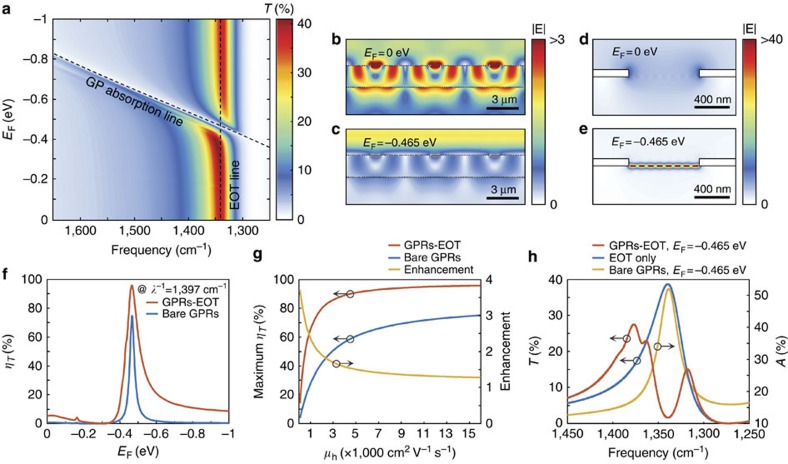
Coupled structure simulations. (**a**) Transmittance map exhibiting graphene plasmon (GP) absorption and extraordinary optical transmission (EOT) as a function of frequency and graphene Fermi level (*E*_F_). (**b**–**e**) Total electric field distributions when turning the graphene plasmons off (*E*_F_=0 eV) and on (*E*_F_=−0.465 eV) at *λ*^−1^=1,340 cm^−1^. (**f**) Comparison of modulation efficiency in transmission (*η*_*T*_) at *λ*^−1^=1,340 cm^−1^ as a function of *E*_F_ between the bare graphene plasmonic ribbons (GPRs) and the GPRs coupled to an extraordinary optical transmission structure (GPRs–EOT). (**g**) Maximum modulation efficiency in transmission of bare GPRs and GPRs–EOT as a function of graphene carrier mobility (*μ*_h_). The enhancement factor is calculated from the ratio of the maximum modulation efficiencies. (**h**) Anti-crossing behaviour (red line) by strong coupling between the EOT resonance without GPRs (blue line) and the resonance in bare GPRs (yellow line).

In the proposed coupled structure, GPRs are located inside subwavelength metallic slits to modulate the coupling between the surface plasmon modes on the top and bottom metal surfaces. Four parameters dictate the subwavelength metallic slit array design: the metallic material, metal thickness, slit width, and period in transverse direction, as shown in Fig. 1a. We used Au with a thickness of 80 nm, and the subwavelength metallic slit width is 800 nm, which can support eight GPRs inside each metallic slit. For a given subwavelength metallic slit width, the EOT resonance frequency is determined by the subwavelength metallic slit array period. A period of 5.54 μm is used in simulation to match an EOT peak at *λ*^−1^=1,340 cm^−1^. After combining the GPRs and the subwavelength metallic slit array, the 0th order transmittance (*T*) as a function of frequency and *E*_F_ is shown in Fig. 3a. When the plasmonic resonance in GPRs deviates from the EOT resonance frequency, the subwavelength metallic slit array exhibits resonant transmission. However, when the GPRs are gated such that plasmonic resonance in GPRs matches the EOT resonance (*E*_F_=−0.465 eV), there is a strong dip in the transmission spectrum at the crossing between two resonant modes, as shown in Fig. 3a. The total electric field distributions on and off the plasmonic resonance in GPRs are shown in Fig. 3b–e. When the plasmonic resonance in GPRs is detuned from the EOT mode, we observe a metallic surface plasmon mode on the bottom metal surface and confined fields inside the subwavelength metallic slits, indicating coupling between the two surface plasmon modes (Fig. 3b,d). In contrast, as the plasmonic resonance in GPRs is tuned to the EOT frequency (*E*_F_=−0.465 eV), both the metallic surface plasmon modes on the bottom metal surface and inside the subwavelength metallic slits are diminished significantly (Fig. 3c) because the coupling channel is blocked through interaction with the intergap GPRs (Fig. 3e). To evaluate the modulation performance, we compare modulation efficiencies of the bare GPRs and GPRs combined with the subwavelength metallic slit array (GPRs–EOT). Here, the modulation efficiency in transmittance (*η*_*T*_) is defined by 1−*T*/*T*_max_, where *T* is transmittance as a function of *E*_F_, and *T*_max_ corresponds to the transmission spectrum for the graphene Fermi level position that maximizes the transmitted intensity at the resonant frequency. In this simulation for GPRs, *T*_max_ occurs at *E*_F_=−0.310 eV, where there is sufficient doping to prevent graphene inter-band absorption, but insufficient carrier density to support plasmonic modes in the GPRs. Figure 3f shows that, for high carrier mobility graphene (*μ*_h_=15,000 cm^2^ V^−1^ s^−1^), the coupled structure shows a moderate improvement over the bare GPRs, with the theoretical maximum modulation efficiency increasing from *η*_*T*_=74.5–95.7%. As the graphene carrier mobility is lowered, the overall modulation efficiency decreases in both devices. However, the relative benefits of the GPRs–EOT structure are enhanced with lower graphene carrier mobility, as shown in Fig. 3g (also see Supplementary Fig. 2). For example, at *μ*_h_=3,000 cm^2^ V^−1^ s^−1^ the modulation efficiency of the bare GPRs is 50.6%, while the GPRs-EOT structure achieves 85.9% efficiency, and for *μ*_h_=1,000 cm^2^ V^−1^ s^−1^ these values change to 27.3% and 65.2%, respectively. Thus the GPRs-EOT structure can exhibit large transmission modulation and is more robust against ribbon disorder compared with bare GPR-based devices.

Modulation can be interpreted qualitatively using an effective medium theory that considers plasmonic modes in the subwavelength metallic slit array coupled to deeply subwavelength GPRs. There is significant spatial overlap between the plasmonic modes in GPRs and the EOT modes of the subwavelength metallic slit array, such that plasmonic resonances in GPRs can alter the local dielectric environment experienced by EOT modes when the two frequencies approach one another. For *E*_F_ values far above or far below −0.465 eV, resonant absorption of the GPRs is far away from the transmission resonance of the subwavelength metallic slit array, and the two effects effectively behave independently. As *E*_F_ approaches −0.465 eV, however, the plasmonic resonance in GPRs creates large deviations in the local dielectric function near the energy of the EOT mode. Specifically, at energies just below or above the plasmonic resonance in GPRs, the effective permittivity is increased or decreased, respectively. This allows the subwavelength metallic slit array to support two distinct modes, even though its geometry selects for only one wave vector. That is, a longer wavelength mode exists which experiences a larger permittivity, and a shorter wavelength mode exists which experiences a smaller permittivity. This creates the splitting, or anti-crossing behaviour between the graphene plasmonic resonant mode and the EOT resonant mode, as observed in Fig. 3h (also see Supplementary Note 1), and the coupling strength between the EOT structure and the embedded GPRs can be determined from the frequency splitting.

As shown in Fig. 3h, the frequency splitting is larger than the linewidth of each of the two resonant modes. This indicates that the energy exchange rate between the two resonant modes is faster than the damping rate of each mode, which suggests that the GPRs–EOT device is operating in the strong coupling regime^34^. The strong coupling nature of this device is further confirmed by modelling the frequency splitting as a function of graphene ribbon density, from which we find a square root relationship^35^,^36^ (Supplementary Fig. 3). As the graphene carrier mobility is lowered, the *Q*-factor of the GPRs decreases, and this anti-crossing behavior is lost^34^ (Supplementary Fig. 4). In this regime (with *μ*_h_≤1,000 cm^2^ V^−1^ s^−1^), the GPRs–EOT modulator still displays relatively high efficiencies, but the modulation is achieved by absorption in the GPRs, where the optical modes decay too rapidly to interact strongly with the EOT structure. Notably, the modulation enhancement of the GPRs–EOT device over the bare GPRs device becomes more significant in this damping-dominant regime. Therefore we suggest that coupling between the two resonant modes is the dominant mechanism for light modulation for high carrier mobility graphene (or equivalently a high *Q*-factor in the graphene plasmonic resonant mode), while the GPRs with low carrier mobility graphene simply damp the EOT mode.

### Mid-infrared transmission measurement

To demonstrate the modulator performance, we fabricated bare GPRs and GPRs–EOT on 2 μm SiN_*x*_ membranes. First, a transparent back gate electrode composed of 4 nm of ITO and 60 nm of a-Si was sputtered onto the bottom side of the SiN_*x*_ membrane. Since this back electrode is directly connected to the Si frame of the SiN_*x*_ membrane, we can apply a gating voltage through the Si frame^10^,^17^,^19^. After transferring CVD-grown graphene to the top surface (see Methods, Supplementary Note 2 and Supplementary Fig. 5), the GPRs were patterned using e-beam lithography followed by reactive ion etching. Finally, we defined subwavelength metallic slit arrays by electron beam lithography and metal evaporation (Ti 3 nm, Au 80 nm). The slit width is 800 nm, and the period of the slit array is 5.6 μm, which puts the EOT peak at *λ*^−1^=1,403 cm^−1^ with undoped graphene in a mid-infrared transmission measurement. Transmission measurements were performed using a Fourier transform infrared (FTIR) microscope with a polarizer to eliminate the transverse electric component from the incoming light.

**Figure 4:**
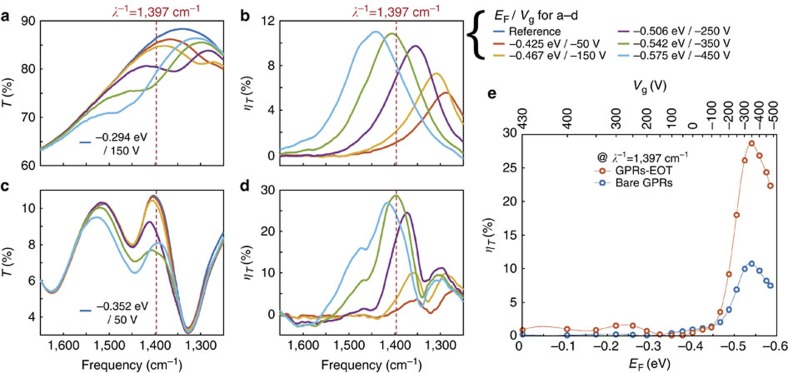
Experimental results. (**a**) Measured transmission spectra and (**b**) modulation efficiency (*η*_*T*_) of the bare graphene plasmonic ribbons (GPRs) device for varying graphene Fermi level *E*_F_ (or gate voltage *V*_g_). (**c**) Measured transmission spectra and (**d**) modulation efficiency (*η*_*T*_) of the graphene plasmonic ribbons coupled to extraordinary optical transmission structure (GPRs–EOT) device for varying graphene Fermi level *E*_F_ (or gate voltage *V*_g_). The legends for **a**–**d** are shown on the right side of **b**. (**e**) Measured modulation efficiency comparison at *λ*^−1^=1,397 cm^−1^ as a function of *E*_F_ between bare GPRs device and GPRs–EOT device.

Figure 4 compares the experimentally measured transmission spectra and modulation features of bare GPRs and GPRs–EOT devices. As shown in Fig. 4a,c, both devices display gate-dependent transmission features that become stronger and shift to higher energies with increased graphene doping. To calculate and compare the modulation efficiencies, transmission spectra are normalized by the transmission spectrum with *E*_F_=−0.294 eV for bare GPRs device and *E*_F_=−0.353 eV for GPRs–EOT device, corresponding to graphene Fermi levels that exhibit maximum transmittance at the EOT resonance frequency. The resulting gate-dependent modulation efficiencies in transmission are shown in Fig. 4b,d. Both devices exhibit narrowband modulation features that become more intense and blue shift with higher graphene doping. The subwavelength metallic slit array exhibits an EOT peak at *λ*^−1^=1,403 cm^−1^ where no plasmons exist in the GPRs because of low doping, as shown in Fig. 4c. As doping increases, plasmons are excited in the GPRs inside the subwavelength metallic slits, and block the coupling channel for the EOT resonance. As a result, we observe the transmittance at the EOT peak decline until *E*_F_ reaches −0.542 eV, which corresponds to the crossing point between the EOT resonance and the plasmonic resonance in GPRs. Figure 4e summarizes the modulation efficiencies of bare GPRs and GPRs-EOT devices. At *λ*^−1^=1,397 cm^−1^, the GPRs–EOT device shows maximal modulation efficiency of 28.6% with *E*_F_=−0.542 eV, while the bare GPRs device has a maximum modulation efficiency of only 10.7% at the same *E*_F_.

## Discussion

**Figure 5:**
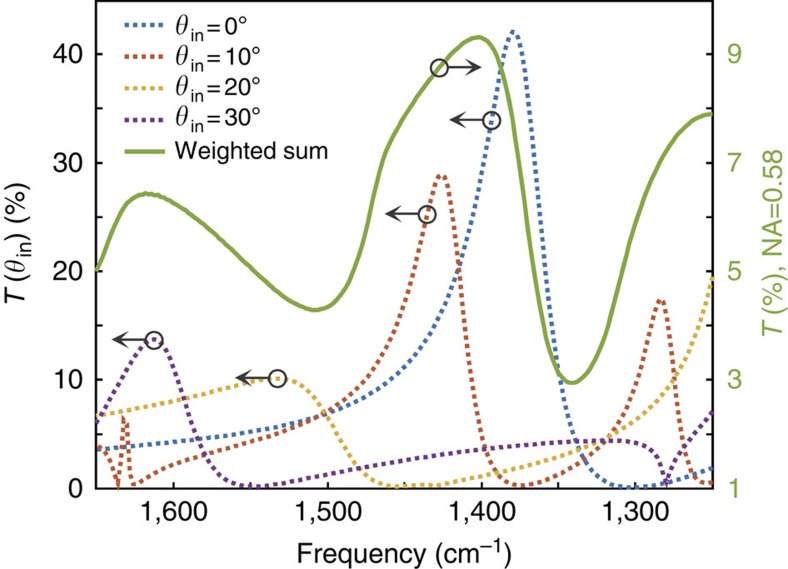
Numerical aperture effect. Simulation results demonstrating the effect of a broad angular distribution of incoming light. Dotted lines (left axis): transmission spectra of the subwavelength metallic slit array with different incident angles (*θ*_in_). Solid line (right axis): weighted sum of transmission spectra at different angles for an objective numerical aperture (NA) of 0.58.

The experimental measurements shown in Fig. 4 differ from simulations in a number of important ways. Notably, the experimental modulation is lower than the simulated one, and the spectral width of the experimental transmission resonance is significantly broader. These features can be attributed to a number of factors that distinguish measurement from simulations. The incoming light is illuminated by a Cassegrain-type objective lens with a high numerical aperture (NA) of 0.58, which means the light incidence angle ranges from −35° to 35°. Such a broad angular distribution of incident light results in a broad transmission spectrum and lower transmittance in the EOT structure because the EOT resonance condition strongly depends on incident angle as well as the period of the subwavelength metallic slit array. To estimate the effect from the broad incidence angular distribution, we calculated transmission characteristics by superposing spectra having the incident angle from −35° to 35° with 1° intervals. In these simulations, the period of the subwavelength metallic slit array is 5.2 μm, matching the EOT resonance frequency (1,403 cm^−1^) in our measurements. The resulting calculated transmission spectra are shown in Fig. 5, and the superposed spectrum (solid green line) shows several differences relative to the transmission spectrum for normally incident light (dotted blue line). Specifically, the superposed spectrum reveals a broader transmission spectrum with an additional peak at higher frequency (1,618 cm^−1^). Moreover, the angular spread of incident light lowers the maximum theoretical transmittance of the extraordinary optical transmission structure from 42.1 to 9.32%.

In addition to the broad light incidence angular distribution, some imperfections in fabrication could degrade the modulation efficiency, including PMMA residue on graphene, carrier density variation in the graphene created by localized charges, a variation in the width of the GPRs resulting from lithography, edge states of the GPRs induced by the etching process, and some resonators that are electronically isolated. These imperfections result in broadening of the graphene plasmon resonance linewidth and a lower modulation efficiency compared with simulations. To account for the broad incident angular distribution and imperfections in fabrication, we tuned graphene carrier mobility and employed a scaling factor as fitting parameters to explain the linewidth and modulation efficiency (see Supplementary Note 3 and Supplementary Fig. 6). Fitting results show that a graphene carrier mobility of 450 cm^2^ V^−1^ s^−1^ is in good agreement with the graphene plasmon resonance linewidth, which indicates that damping was a dominant factor in the light modulation for this experiment. Simulations with scaling factors of 0.633 for the bare GPRs device and 0.734 for the GPRs–EOT device are able to fit the measurement results. With these fitting parameters, the expected modulation efficiencies with purely normally incident light are 11.4% for the bare GPRs device and 36.0% for the GPRs–EOT device.

In contrast to the decreased transmission seen here due to plasmonic absorption in the GPRs, it has been reported that transmission can increase via absorption, described as absorption-induced transparency^37^,^38^,^39^. In ref. 37, a nanodisk is used to extract photons from a subwavelength hole and scatter them into free space, so that light transmission is enhanced. This mechanism is possible because the nanodisk's scattering cross-section is comparable to its absorption cross-section. Refs 38,39^38^,^39^ report that EOT structures hybridized with dye absorber layers show an increase in EOT transmission when the dye is placed inside the subwavelength holes. In those devices, the absorbing medium fills the subwavelength metallic holes, allowing for an altered in-hole propagation constant. While those effects may play some role in the transmission properties of the structure we propose here, we note that in this device the transmission is decreased as the absorbing plasmonic resonances are activated, rather than increased. In the device we demonstrate here, however, the scattering cross-section of GPRs is much smaller than their absorption cross-section due to the extreme spatial confinement^20^. In addition, the GPRs are located only at the bottom of the subwavelength metallic slits, making such an effect less likely. While absorption-induced transparency might be achievable in devices where the array of tunable GPRs completely fills the subwavelength metallic slits, it is difficult to experimentally realize such a device.

In summary, GPRs coupled to subwavelength metallic slit array that exhibit extraordinary optical transmission enable strong transmission modulation at mid-infrared frequencies. Light absorption in GPRs efficiently suppresses the extraordinary optical transmission resonance resulting in high modulation efficiency. Simulations indicate a transmission modulation efficiency of 95.7%. This modulation occurs with small changes in the graphene Fermi level position, within ranges that are accessible with electrostatic gating methods. Experimental mid-infrared transmission measurements of a fabricated device demonstrate that the proposed device exhibits 2.67 times higher modulation efficiency than that of a bare GPRs device. The experimental modulation efficiency could be enhanced by use of graphene with higher carrier mobility and by transmission measurements using a parallel rather than a convergent beam illumination configuration since extraordinary optical transmission resonances are quite angle-sensitive. The results illustrate the potential for coupling graphene resonances and conventional noble metal plasmon resonances to achieve transmission-type light modulation, which may be useful in, for example, actively tunable amplitude modulated infrared metasurfaces and real-time hologram systems.

## Methods

### Material modelling in simulation

The frequency-dependent dielectric function of SiN_*x*_ was measured using mid-infrared ellipsometry (Supplementary Fig. 1b). For ITO, we used tabulated data^40^. In the case of a-Si, we scaled a-Si data from Palik data^41^ by a weighting factor of 0.88, which reflects the optical characteristic difference depending on the sputtering condition, such as temperature and RF power^42^. Supplementary Figure 1a shows that the simulation results and the mid-infrared transmission measurement are in good agreement with these dielectric functions. The dielectric function of Au for the subwavelength metallic slit array structure was taken from Palik data^41^. The dielectric function of graphene is analytically modelled as a thin layer (*t*=0.3 nm) having a permittivity of 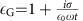. Here the dynamical surface conductivity *σ* is calculated by random phase approximation^20^,^43^.

### CVD graphene growth

CVD graphene was grown on high-purity copper foil (Alfa Aesar, 99.9999%) at 1,000 °C for 1 hour with H_2_ flow rate of 50 sccm and CH_4_ flow rate of 1 sccm (refs 44, 45). After spin-coating PMMA on top, the copper foil was etched using an FeCl_3_ solution (Transene, CE-100). The PMMA/graphene layer was then rinsed in DI water and transferred onto the substrate. Finally, the PMMA layer was removed with acetone. The quality of the CVD-grown graphene was characterized by Raman spectroscopy, where a negligible *D*-band was observed—indicating a low defect density—and a 2*D* to *G* ratio of ∼2 confirmed the monolayer nature of the graphene sheet (Supplementary Fig. 5a). The graphene Fermi level position was determined by measuring the gate-dependent sheet resistance and assigning the resistance maximum as the charge neutral point, where the graphene Fermi level is aligned with the Dirac point. After identifying the charge neutral point (Supplementary Fig. 5b), a simple capacitor model was used to calculate the graphene Fermi level position for a given gate bias^24^.

### Sample fabrication

The DC conducting contact (ITO 4 nm/a-Si 60 nm) was deposited on the bottom side of 2 μm SiN_*x*_ membrane (Norcada, NX10500N) by RF sputtering at room temperature. The RF sputtering powers for the ITO and the a-Si were 48 W and 150 W, respectively. Both GPRs and the subwavelength metallic slit array were patterned by 100 keV electron beam lithography using a PMMA resist. The GPRs were then cut using reactive ion etching with oxygen at 80 W for 15 s, with the patterned PMMA serving as a soft etch mask. The metallic layer was deposited by electron beam evaporation of Ti (3 nm) followed by Au (80 nm).

### Data availability

The authors declare that the data supporting the findings of this study are available within the article and its Supplementary Information files.

## Additional information

**How to cite this article:** Kim, S. *et al.* Electronically tunable extraordinary optical transmission in graphene plasmonic ribbons coupled to subwavelength metallic slit arrays. *Nat. Commun.* 7:12323 doi: 10.1038/ncomms12323 (2016).

## Supplementary Material

Supplementary Information Supplementary Figures 1-6, Supplementary Notes 1-3 and Supplementary References.
