# Supplementary material for: Electronically tunable extraordinary optical transmission in graphene plasmonic ribbons coupled to subwavelength metallic slit arrays
Source: Nat Commun. 2016 Aug 8;7:12323. doi: 10.1038/ncomms12323 (PMC4979088; doi:10.1038/ncomms12323)
Supplement: Supplementary Information — Supplementary Figures 1-6, Supplementary Notes 1-3 and Supplementary References. [file ncomms12323-s1.pdf]

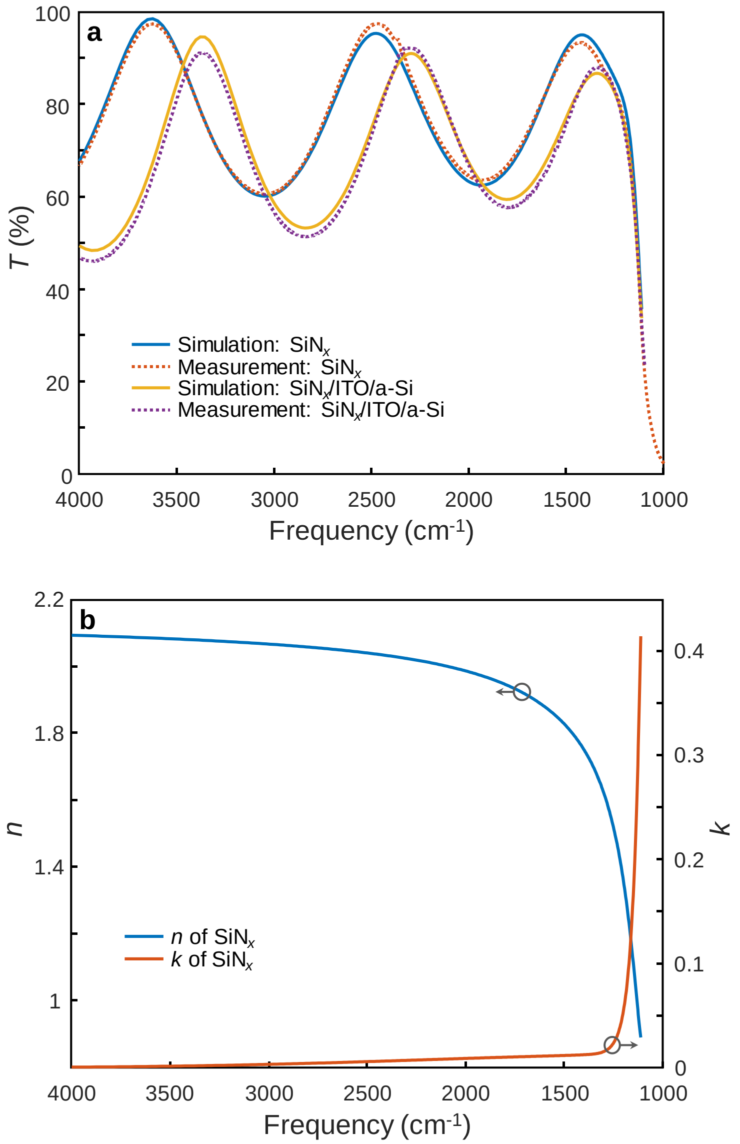

Supplementary Figure 1 | Characteristic of membrane. (**a**) Transmission spectra for a single SiN_x 2μm membrane and a SiN_x membrane with transparent back contact (ITO 4nm / a-Si 60nm). The solid lines are simulation results, and the dotted lines were obtained by a mid-infrared transmission measurement. (**b**) Refractive index of SiN_x measured by mid-infrared ellipsometry.

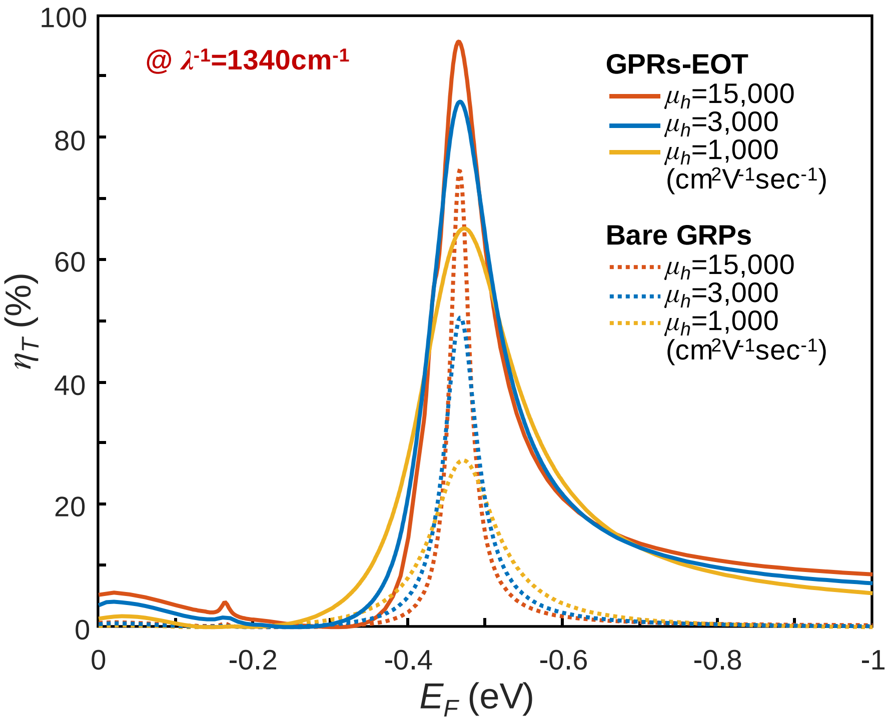

Supplementary Figure 2 | Modulation efficiency in transmission. Comparison of modulation efficiency in transmission (η_T) between the bare graphene plasmonic ribbons (GPRs) device and the coupled structure (GPRs-EOT) device as a function of graphene Fermi level (E_F) with different graphene carrier mobilities (μ_h).

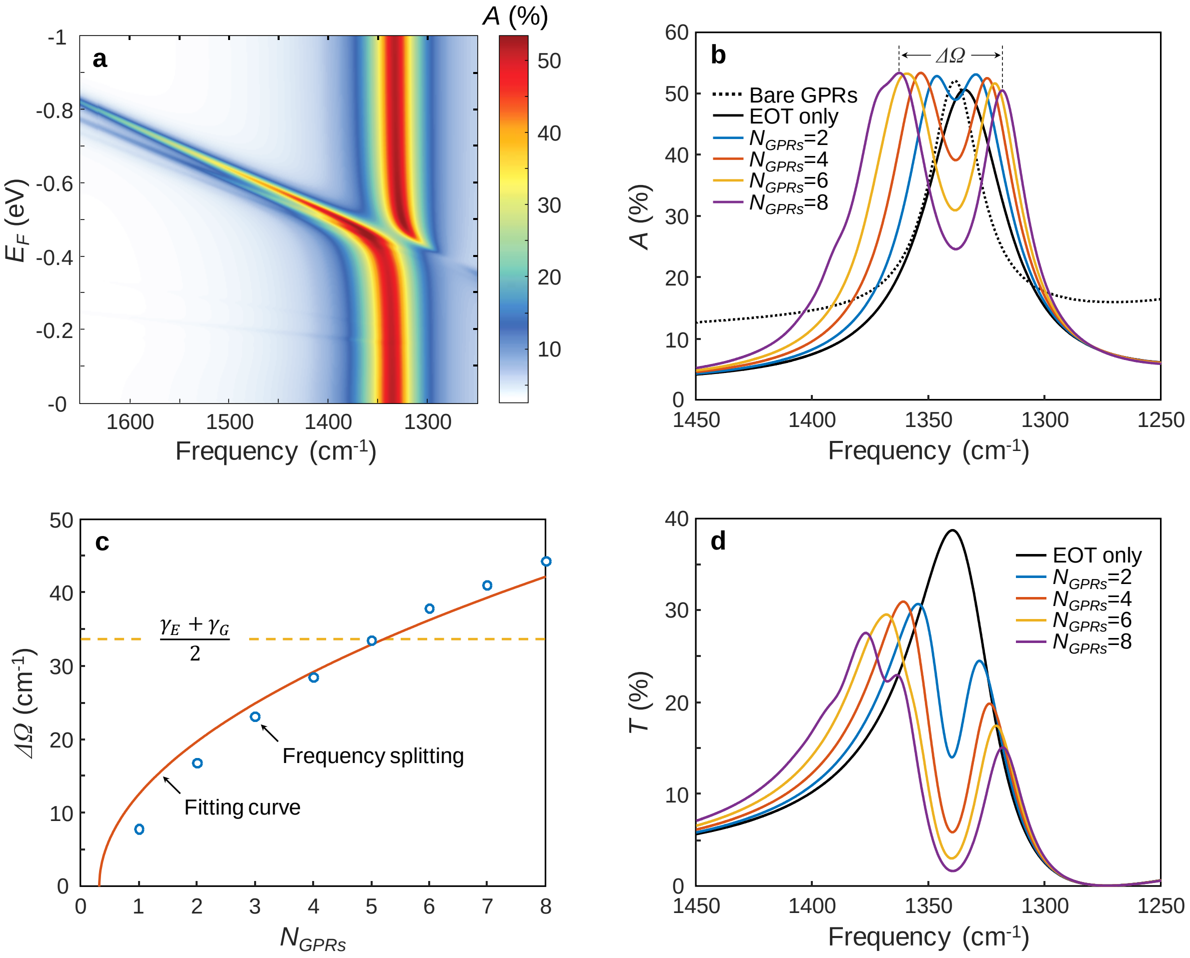

Supplementary Figure 3 | Anti-crossing behavior in the coupled structure. (**a**) Absorption map of the coupled structure (GPRs-EOT) as a function of frequency and graphene Fermi level (E_F) exhibiting anti-crossing behavior. (**b**) Absorption spectra, (**c**) frequency splitting, and (**d**) transmission spectra as a function of number of graphene plasmonic ribbons (N_{GPRs}) inside the subwavelength metallic slit. In (**b**) and (**d**), the "EOT only" indicates the subwavelength metallic slit array without GPRs.

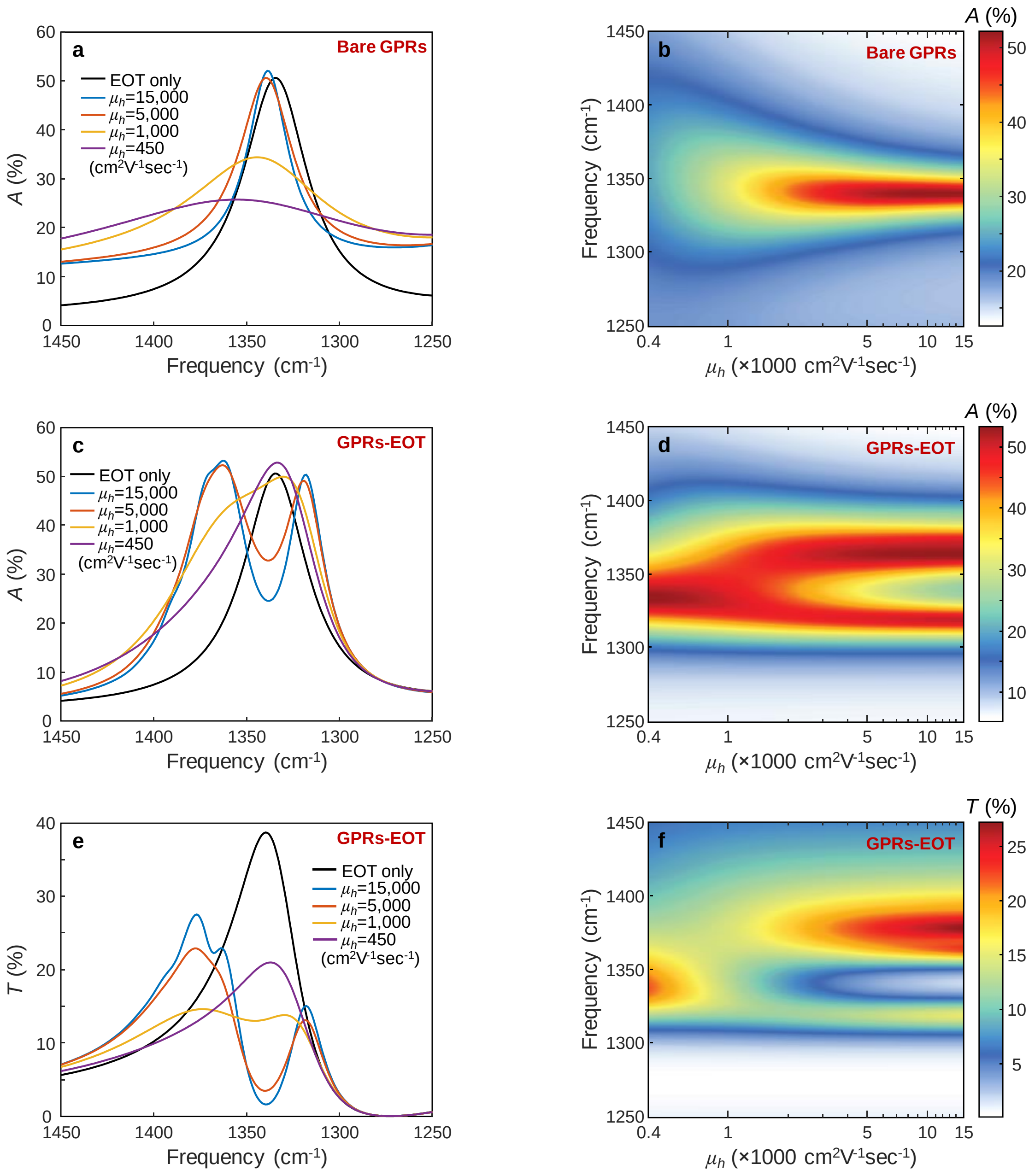

Supplementary Figure 4 | Anti-crossing behavior dependence on graphene carrier mobility. (**a**) Absorption spectra and (**b**) absorption map of the bare graphene plasmonic ribbons (GPRs) device as a function of graphene carrier mobility (μ_h). (**c**) Absorption spectra and (**d**) absorption map of the coupled structure (GPRs-EOT) device as a function of graphene carrier mobility (μ_h). (**e**) Transmission spectra and (**f**) transmission map of the coupled structure (GPRs-EOT) device as a function of graphene carrier mobility (μ_h). In (**a**), (**c**), and (**e**), the "EOT only" indicates the subwavelength metallic slit array without GPRs.

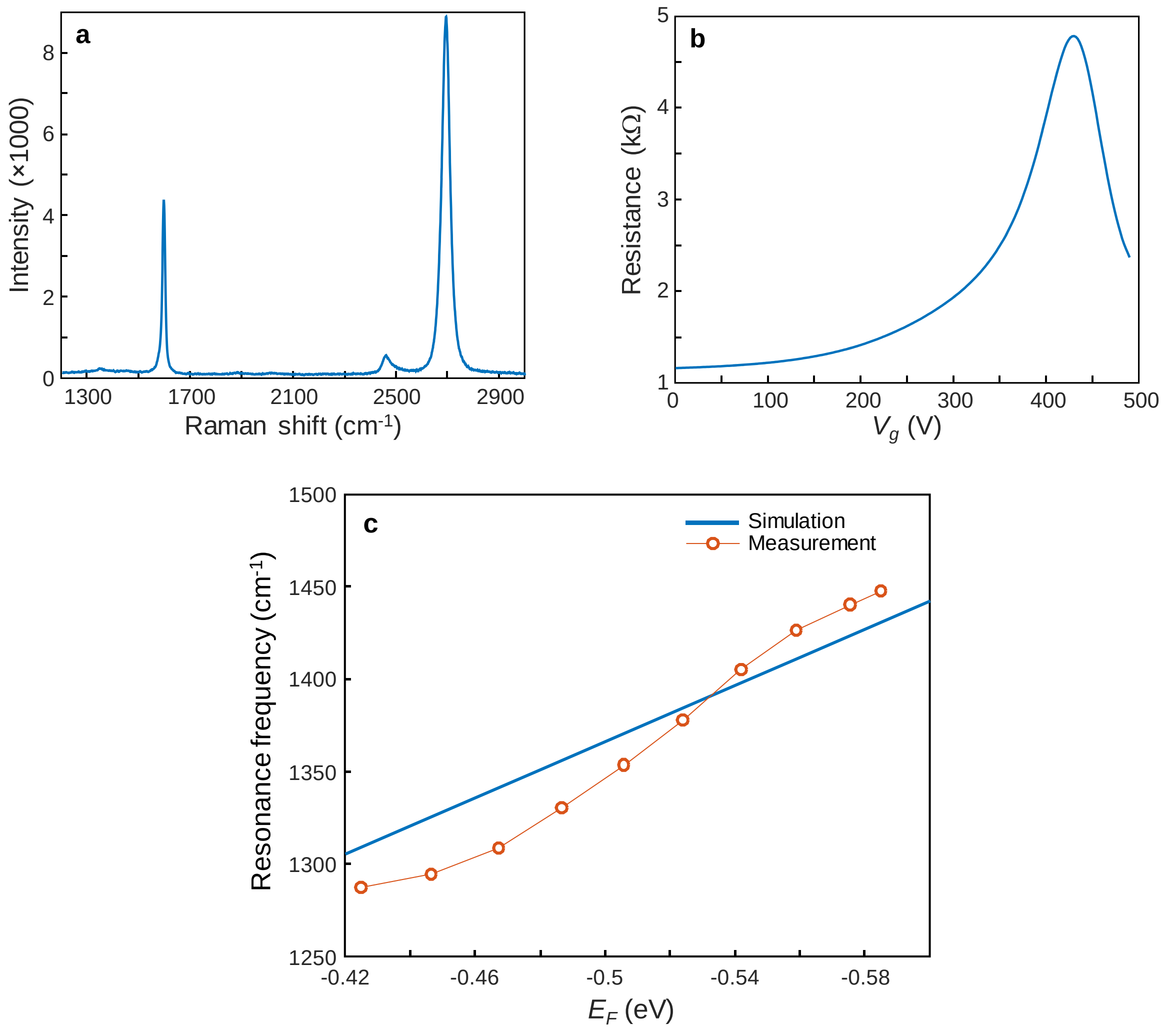

Supplementary Figure 5 | Characteristics of graphene. (**a**) Raman spectrum of graphene transferred onto SiO_2 substrate, and (**b**) gate dependent resistance measurement of graphene on the SiN_x substrate showing the charge neutral point at gating voltage V_g=430V. (**c**) Graphene plasmon resonance frequency as a function of graphene Fermi level (E_F) for simulations and mid-infrared transmission measurement.

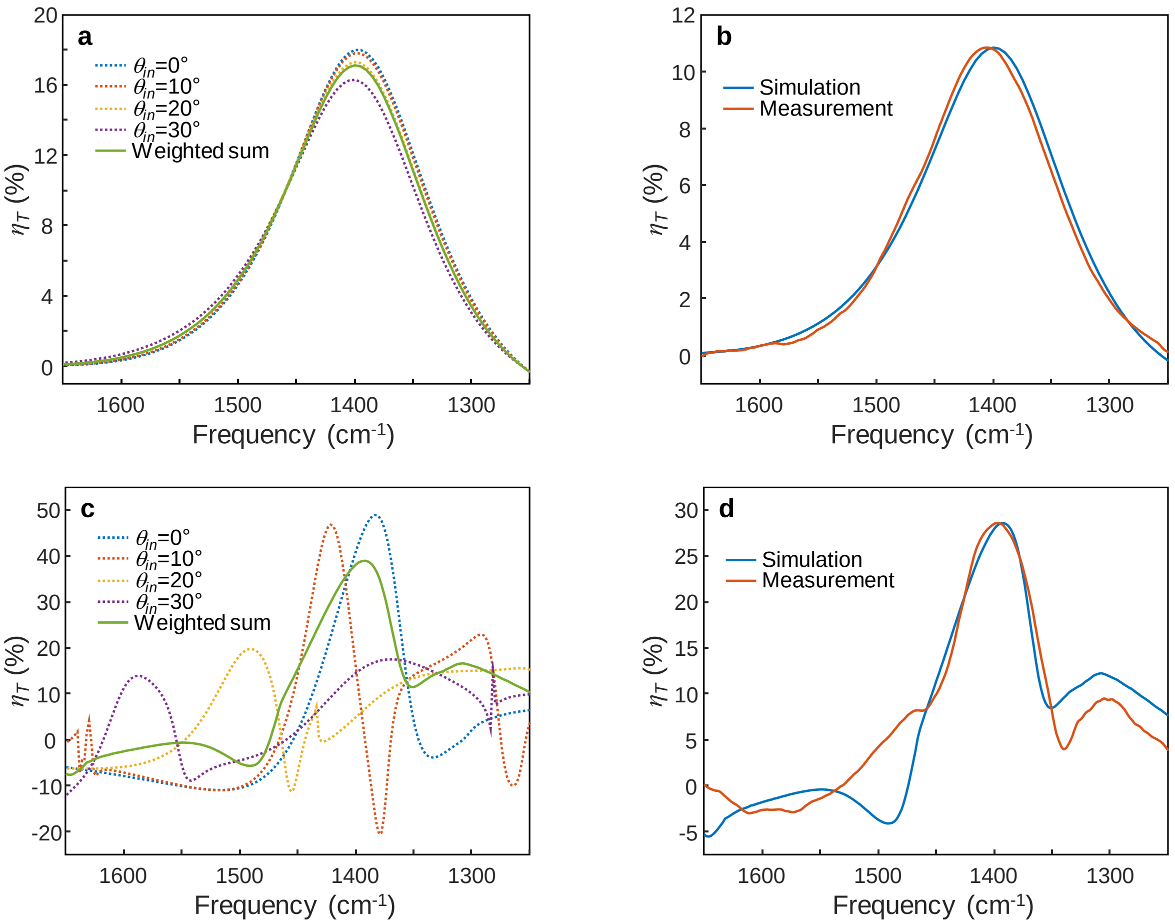

Supplementary Figure 6 | Numerical fitting. (**a**) Calculated modulation efficiencies for bare graphene plasmonic ribbons (GPRs) device varying the incident angle (θ_{in}) and the weighed sum. (**b**) Comparison between the measurement result and the simulation result of bare GPRs with a broad angular distribution of incoming light and a scaling factor of 0.633 to account for degradation. (**c**) Calculated modulation efficiencies for coupled structure (GPRs-EOT) device varying the incident angle (θ_{in}) and the weighed sum. (**d**) Comparison between measurement and simulation result of GPRs-EOT device with a broad angular distribution of incoming light and a scaling factor of 0.734 to account for degradation.

Supplementary Note 1. Anti-crossing behavior in the coupled structure

As shown in Supplementary Fig. 3a, the coupled structure shows anti-crossing behavior at a crossing between the graphene plasmonic resonant mode and extraordinary optical transmission (EOT) resonant mode. We also observed that the frequency splitting depends on the number of graphene plasmonic ribbons (GPRs) inside the subwavelength metallic slit (N_{GPRs})[1,2], as shown in Supplementary Fig. 3b. In this calculation, the pitch of the ribbons was determined by 800nm divided by N_{GPRs}, where the 800nm corresponds to the subwavelength metallic slit width, and the graphene Fermi level for each N_{GPRs} was tuned to minimize the transmission. To evaluate this relationship, we used the classical oscillator model[2]

$$\Delta\Omega \cong \frac{\alpha}{\lambda_{res}^{-1}}\sqrt{N_{GPRs} - \left[\frac{\lambda_{res}^{-1}(\gamma_G - \gamma_E)}{2\alpha}\right]^2} \quad (1)$$

$\Delta\Omega$=frequency splitting, α=coupling coefficient, $\lambda_{\mathrm{res}}^{-1}$=resonance frequency

N_{GPRs}=number of GPRs, γ_G & γ_E =absorption linewidth of bare GPRs and GPRs-EOT, respectively

In this model, we fitted our data of frequency splitting as a function of number of graphene ribbons N_{GPRs} to extract the coupling coefficient α. The best fit was obtained with α=2.04×10^4cm^{-2}, and the root mean square error was 2.39cm^{-1}, as shown in Supplementary Fig. 3c. We believe that the small deviation between the model and the calculated frequency splitting comes from assumptions in the classical oscillator model. The model assumes that a single GPR does not interact with adjacent GPRs, and the coupling coefficient is identical for all GPRs. In a real system, the graphene plasmons are a collective oscillation, which would affect the linewidth γ_G. In addition, the coupling coefficient α would be altered depending on the position of each GPR. Regardless of discrepancies between the assumptions in the model and the real system, this model shows very good agreement with the calculated frequency splitting. In addition, the coupled system exhibits a strong coupling when it contains six or more GPRs, as we can see by considering the frequency splitting and the average linewidth of the two resonant modes, as shown in Supplementary Fig. 3c. As a result of this coupling between two resonant modes, the splitting is also exhibited in transmission spectra, as shown Supplementary Fig. 3d.

To create strong coupling, the energy exchange rate should be faster than the decay rate of each resonant mode[3]. Therefore, the anti-crossing behavior disappears if the Q-factor of one resonant mode becomes too low, which happens with a low graphene carrier mobility. As shown in Supplementary Figs. 4a and b, the Q-factor of the GPRs becomes lower as the graphene carrier mobility is decreased. As a

result, the anti-crossing behavior in the coupled structure is nearly indiscernible at μ_h=1,000cm^{2}V^{-1}sec^{-1}, and completely disappears at μ_h=450cm^{2}V^{-1}sec^{-1}, as shown in Supplementary Figs. 4c and d. At a low graphene carrier mobility, there is no dip in the absorption spectra. A clear dip in the absorption spectra begins to emerge at μ_h=1,500cm^{2}V^{-1}sec^{-1}, and the frequency splitting is nearly saturated above μ_h=5,000cm^{2}V^{-1}sec^{-1}. This tendency is also observed in the transmission spectra, as shown in Supplementary Figs. 4e and f.

Supplementary Note 2. Characteristics of CVD-grown graphene and determination of graphene Fermi level

Silicon nitride (SiN_x) exhibits photoluminescence (PL) emission over the visible range[4]. Since the PL signal is much stronger than the Raman signal from graphene, the Raman peaks are almost indiscernible when the graphene is transferred onto the SiN_x membrane. Therefore, we measured the Raman spectrum after transferring the CVD-grown graphene onto SiO_2 substrate, as shown in Supplementary Fig. 5a. The G-peak and the 2D-peak are located at 1595cm^{-1} and 2694cm^{-1}, respectively, and their ratio of I_{2D}/I_G=2.04. The Raman spectrum shows that the D-peak (1348cm^{-1}), which corresponds to defects in graphene, is very small, and the ratio of I_G/I_D=17.9.

To calculate the graphene Fermi level of graphene on SiN_x membrane from the gate voltage (V_g) between the graphene and the back contact, we used a capacitor model[5] based on the charge neutral point (CNP) measured by a gate dependent resistance measurement of graphene[6,7], as shown in Supplementary Fig. 5b. In the calculation, we assumed the dielectric constant of SiN_x as 10 (Supplementary Ref. 6). As shown in the Supplementary Fig. 5c, the graphene plasmon resonance frequency depending on graphene Fermi level between simulations and mid-infrared transmission measurement shows good agreement with this dielectric constant. The slight discrepancy between the simulation and the measurement results could come from atmospheric and substrate impurities[8,9]. Non-uniform DC electric field along the graphene ribbons, such as the lightning rod effect at the edges, could also affect the doping level.

Supplementary Note 3. Numerical fitting of the simulation with measured transmission spectra

A real experiment differs from simulations in several ways. First, the finite numerical aperture (NA) of the objective lens used in Fourier transform infrared (FTIR) microscope induces a broad angular

distribution in incoming light in contrast to purely normal incident light used in simulations. Second, some imperfections in fabrication could lower the graphene quality. Such factors could cause broad linewidth in the modulation spectrum and low modulation efficiency. To take into account these factors, we employed a low graphene carrier mobility to fit the linewidth, and a scaling factor to compensate the modulation efficiency[6].

When it comes to the finite NA (0.58) of the objective lens, we simulated the structure varying the incident angle from -35° to 35° with 1° step. In the case of bare GPRs, the simulation results show that the NA effect is not so large, as shown in Supplementary Fig. 6a. Although the modulation efficiency decreases slightly as the incident angle increases, the line shape or the peak position do not change significantly. In this simulation, we used a graphene carrier mobility of 450cm^{2}V^{-1}sec^{-1}, which results in good agreement between the simulation and measurement results in terms of linewidth of the modulation spectrum.

Supplementary Figure 6b shows the simulation and experimental data with E_F=-0.542eV, which corresponds to the graphene Fermi level showing the maximum modulation efficiency in the coupled structure (GPRs-EOT) device. With aforementioned broad angular distribution of incoming light, a graphene carrier mobility of 450cm^{2}V^{-1}sec^{-1} and a scaling factor of 0.633 to account for degradation, the simulation result matches the measurement result very well.

In contrast to the bare GPRs, the broad angular distribution of incoming light significantly affects the GPRs-EOT device because the EOT resonance itself strongly depends on the incident angle, as shown in Fig. 5. Such a strong dependence of modulation is shown in Supplementary Fig. 6c. In this simulation, we used the same graphene carrier mobility of bare GPRs. Similar to the EOT spectrum in Fig. 5, the modulation peak also blue-shifts with oblique incident light. As a result, the maximum modulation efficiency of the weighted sum spectrum is reduced by 20.3% compared with the modulation spectrum using purely normal incoming light.

In Supplementary Fig. 6d, we compared the measurement data with simulation results with a scaling factor of 0.734 to account for degradation. This value is slightly higher compared to the scaling factor for bare GPRs. We expect that there are less dead resonators in the GPRs inside the subwavelength metal slits than in the bare GPRs device because the dimension in transverse direction is much shorter compared with the bare GPRs structure, and therefore could reduce the chance of disconnection.

Supplementary References

1. Agarwal, G. S. Vacuum-field Rabi splittings in microwave absorption by Rydberg atoms in a cavity. *Phys. Rev. Lett*. **53**, 1732-1734 (1984).
2. Rudin, S. & Reinecke, T. L. Oscillator model for vacuum Rabi splitting in microcavities. *Phys. Rev. B* **59**, 10227-10233 (1999).
3. Khitrova, G., Gibbs, H. M., Kira, M., Koch, S. W. & Scherer, A. Vacuum Rabi splitting in semiconductors. *Nat. Phys*. **2**, 81-90 (2006).
4. Kistner, J. *et al*. Photoluminescence from silicon nitride-no quantum effect. *J. Appl. Phys*. **110**, 023520 (2011).
5. Das, A. *et al*. Monitoring dopants by Raman scattering in an electrochemically top-gated graphene transistor. *Nat. Nanotechnol*. **3**, 210-215 (2008).
6. Jang, M. S. *et al*. Tunable large resonant absorption in a midinfrared graphene Salisbury screen. *Phys. Rev. B* **90**, 165409 (2014).
7. Brar, V. W. *et al*. Electronic modulation of infrared radiation in graphene plasmonic resonators. *Nat. Commun*. **6**, 7032 (2015).
8. Levesque, P. L. *et al*. Probing Charge Transfer at Surfaces Using Graphene Transistors. *Nano Lett*. **11**, 132-137 (2011).
9. Ryu, S. *et al*. Atmospheric Oxygen Binding and Hole Doping in Deformed Graphene on a SiO_2 Substrate. *Nano Lett*. **10**, 4944-4951 (2010).
